# Electrical spin injection and detection in molybdenum disulfide multilayer channel

**DOI:** 10.1038/ncomms14947

**Published:** 2017-04-07

**Authors:** Shiheng Liang, Huaiwen Yang, Pierre Renucci, Bingshan Tao, Piotr Laczkowski, Stefan Mc-Murtry, Gang Wang, Xavier Marie, Jean-Marie George, Sébastien Petit-Watelot, Abdelhak Djeffal, Stéphane Mangin, Henri Jaffrès, Yuan Lu

**Affiliations:** 1 Institut Jean Lamour, UMR 7198, CNRS-Université de Lorraine, BP 239, 54506 Vandœuvre, France; 2 Université de Toulouse, INSA-CNRS-UPS, LPCNO, 135 avenue de Rangueil, 31077 Toulouse, France; 3 Unité Mixte de Physique, CNRS, Thales, Univ. Paris-Sud, Université Paris-Saclay, 91767 Palaiseau, France

## Abstract

Molybdenum disulfide has recently emerged as a promising two-dimensional semiconducting material for nano-electronic, opto-electronic and spintronic applications. However, the demonstration of an electron spin transport through a semiconducting MoS_2_ channel remains challenging. Here we show the evidence of the electrical spin injection and detection in the conduction band of a multilayer MoS_2_ semiconducting channel using a two-terminal spin-valve configuration geometry. A magnetoresistance around 1% has been observed through a 450 nm long, 6 monolayer thick MoS_2_ channel with a Co/MgO tunnelling spin injector and detector. It is found that keeping a good balance between the interface resistance and channel resistance is mandatory for the observation of the two-terminal magnetoresistance. Moreover, the electron spin-relaxation is found to be greatly suppressed in the multilayer MoS_2_ channel with an in-plane spin polarization. The long spin diffusion length (approximately ∼235 nm) could open a new avenue for spintronic applications using multilayer transition metal dichalcogenides.

## Abstract (web summary)

MoS_2_ is a promising two-dimensional candidate for opto-electronic and spintronic applications. Here, the authors report electrical spin injection and detection in a few-layered MoS_2_ channel, demonstrating that the spin diffusion length is at least 235 nm in MoS_2_ conduction band.

Transition metal dichalcogenides (TMDs) have emerged as a promising 2D crystal family, demonstrating solutions for several novel nano-electronic and opto-electronic applications^1^,^2^,^3^,^4^,^5^,^6^,^7^. In contrast to graphene and boron nitride, which are respectively a metal and a wide-gap semiconductor, TMDs family displays a large variety of electronic properties ranging from semiconductivity to superconductivity^8^. As a representative of TMDs, molybdenum disulfide (MoS_2_) has a tunable bandgap that changes from an indirect gap of 1.2 eV in the bulk to a direct gap of 1.8 eV for one monolayer (ML)^1^. The ML MoS_2_ is characterized by a large spin-orbit splitting of ∼0.15 eV in the valence band^3^,^4^ together with a small value of ∼3 meV for the conduction band^9^. The lack of inversion symmetry combined with the spin-orbit interaction leads to a unique coupling of the spin and valley degrees of freedom, yielding robust spin and valley polarizations^4^,^5^,^6^,^7^.

To realize electron spin transport in the vicinity of the conduction band of MoS_2_ channel, one of the prerequisites is the investigation of the electron spin-relaxation mechanism within the host material. For ML MoS_2_, both the intrinsic spin splitting of the valence band and the Rashba-like spin-orbit coupling (SOC) due to the breaking of the inversion symmetry along the growth direction favour the spin transport through MoS_2_ with an out-of-plane spin polarization^10^. This is because that the SOC creates an equivalent perpendicular *k*-dependent magnetic field due to the Dresselhaus effective interactions and it is associated to the D'yakonov-Perel (DP) spin relaxation mechanism^11^. If electrons with in-plane spin polarization are injected into ML MoS_2_, the effective magnetic field can induce an efficient in-plane spin precession along the field^12^ as well as the spin-dephasing. Consequently, this yields a predicted short spin lifetime (10–200 ps)^13^ together with a small spin diffusion length (∼20 nm)^14^. Recently, a hole spin injection into ML TMDs has been demonstrated either with perpendicular magnetized GaMnAs injector^15^ or NiFe injector at large perpendicular magnetic field^16^ by electrical injection and optical detection method. This particularly emphasizes on the difficult issue to electrically inject and detect electron with in-plane spin polarization in a lateral ML MoS_2_ device. To avoid the DP spin relaxation, one solution is to recover the inversion symmetry with thicker multilayer MoS_2_. The recent measurement of the second-harmonic generation (SHG) efficiency as a function of the number of MLs is a good probe of the TMDs material symmetry^17^. For one ML, a strong SHG is detected due to the lack of inversion symmetry^18^. However, in the case of bilayers and 4ML MoS_2_, the magnitude of SHG signal decreases by three orders of magnitude due to the recovery of inversion symmetry. Longer spin relaxation time can be expected for such structures. Thus, we consider only multilayer MoS_2_ to demonstrate the in-plane electrical spin injection and detection.

Here, we provide a clear demonstration of a robust spin-valve magnetoresistance (MR) (1.1%) through the conduction band of 6ML thick MoS_2_ channel with ferromagnetic (FM) Co/MgO tunnel injector at low temperature. This occurs in the optimal experimental situation of impedance matching between the interface resistance and the channel resistance. The clear spin-injection signals in MoS_2_ demonstrate a spin-transport in MoS_2_ with a relative long spin-diffusion length larger than 200 nm. This could open future avenues to use multilayer TMDs as an in-plane spin transport template and the electron spin can be manipulated by SOC for a well-defined MoS_2_ thickness controlled by plasma etching technique^19^.

## Results

### MoS_2_ contact and channel resistance

A key issue for electrical spin injection is the conductivity mismatch between the FM injector and the semiconducting MoS_2_ channel, which generally results in a vanishing MR^20^,^21^ due to the spin-backflow processes^21^. In FM/MoS_2_ contacts, a Schottky barrier (SB) height (Φ_b_) of 100–180 meV is generally created at the interface with an extended depletion region^22^,^23^. However, it has been recently demonstrated that an efficient reduction of Φ_b_ down to ∼10 meV at zero back-gate voltage can be achieved by inserting a 1–2 nm layer of MgO (ref. 23), Al_2_O_3_ (ref. 24) or TiO_2_ (ref. 25) as a thin tunnel barrier between the FM and MoS_2_. A careful design of the interface structure consisting of an oxide tunnel barrier injector (MgO) on top of an unavoidable Schottky contact thus appears mandatory to get efficient electrical spin injection and spin-detection^26^.

**Figure 1:**
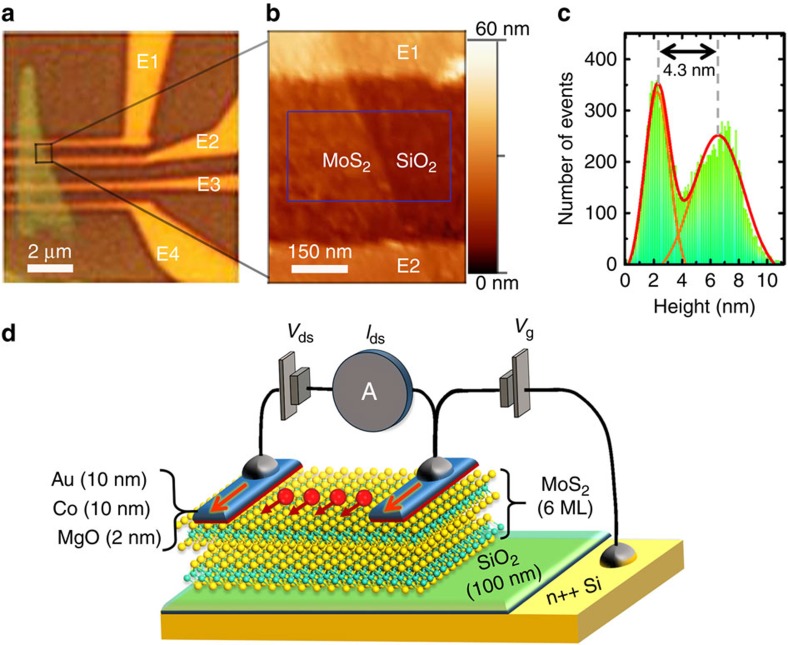
Multilayer MoS_2_-based lateral spin-valve device. (**a**) Optical image of the device with the multilayer MoS_2_ flake on 100 nm SiO_2_/Si(n++) substrate, the E1, E2, E3 and E4 indicate the four Au/Co/MgO electrodes. (**b**,**c**) AFM measurement (in tapping mode) focused on the MoS_2_ channel between E1 and E2 electrodes. The thickness of MoS_2_ is determined by the Gaussian distribution of pixel height in the square region in **b**. (**d**) Schematics of the lateral spin-valve device. The multilayer MoS_2_ serves as a spin transport channel, and two Au/Co/MgO electrodes are used to inject spin (*V*_ds_) and measure the current (*I*_ds_). A back-gate voltage (*V*_g_) between the substrate and one top contact is used to modulate the carrier density in the MoS_2_ channel.

In our devices, MoS_2_ flakes were mechanically exfoliated onto a SiO_2_/Si (n++) substrate. Four FM contacts composed of MgO (2 nm)/Co (10 nm)/Au (10 nm) were deposited on one MoS_2_ flake (see details in Methods). The four electrodes have almost identical width around 300 nm with channel distances varying from 450 to 2,800 nm (Fig. 1a). The thickness of the flake is determined by atomic force microscopy characterization to be about 4.3 nm (Fig. 1b,c). Considering 0.72 nm for one ML MoS_2_ (ref. 27), the thickness of the flake corresponds to 6ML MoS_2_. Figure 1d shows schematics of the device. A drain-source bias (*V*_ds_) between two top contacts was applied to inject a current *I*_ds_. Meanwhile, a back-gate voltage (*V*_g_) was applied between the substrate and one top contact to modulate the carrier density in the MoS_2_ channel.

**Figure 2:**
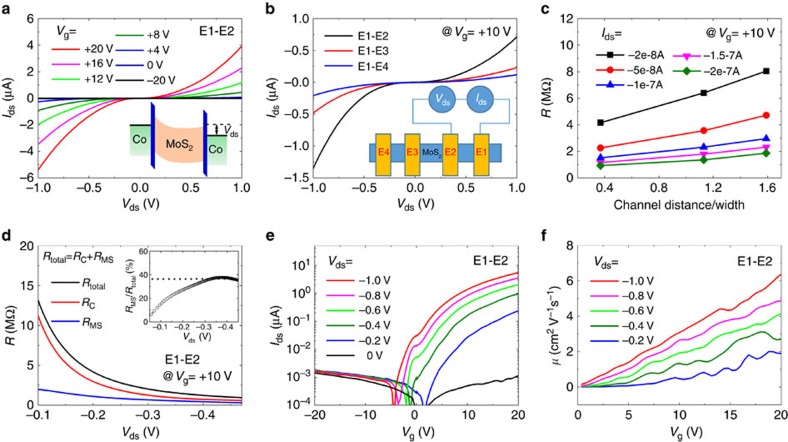
Transport characterization of MoS_2_ field-effect transistor. (**a**) Current (*I*_ds_)–voltage (*V*_ds_) characteristics between the E1 and E2 electrodes, measured at 12 K with applying different back-gate voltages *V*_g_. Inset: band diagram of the back-to-back diode structure of the MoS_2_ device with a two-terminal bias *V*_ds_. (**b**) *I*_ds_–*V*_ds_ characteristics measured between different electrodes with a back-gate voltage *V*_g_=+10 V at 12 K. Inset: schematics of connection between different electrodes. (**c**) The total resistance (*R*) between the two electrodes versus the channel distance (normalized by the width) with different *I*_ds_. (**d**) *V*_ds_ dependence of the total resistance (E1–E2) and the extracted contact resistance *R*_C_ and the MoS_2_ channel resistance *R*_MS_ (E1–E2). Inset: The contribution of *R*_MS_ in the total resistance as a function of *V*_ds_. (**e**)Transfer characteristic *I*_ds_–*V*_g_ between E1 and E2 electrodes, measured at 12 K with applying different *V*_ds_. (**f**) Extracted effective mobility *μ* versus *V*_g_ with different *V*_ds_.

Let us first focus on the two-terminal *I*_ds_–*V*_ds_ characteristics at 12 K between electrodes E1 and E2 with different *V*_g_ (Fig. 2a). At *V*_g_=0 V, the current level is rather low (*I*_ds_∼−100 nA at *V*_ds_=−1 V). By applying a back-gate voltage, a large increase of the current is observed with positive *V*_g_, while the current density is greatly suppressed at negative *V*_g_. The quasi-symmetric nonlinearity of *I*_ds_–*V*_ds_ is attributed to the back-to-back Schottky diode structures of the device (inset of Fig. 2a), thus indicating the important role played by the Co/MgO/MoS_2_ Schottky contacts. The contact region characterized by the contact resistance (*R*_C_) is constituted by the MgO tunnel barrier and the depletion zone of MoS_2_ underneath. To extract the respective contribution of the contact resistance and MoS_2_ channel resistance, we have acquired *I*_ds_–*V*_ds_ curves at *V*_g_=+10 V with different channel distances (Fig. 2b). Those emphasize on a larger contribution of *R*_C_ in the total resistance (*R*_total_) with a shorter channel distance. Since the resistance of the MoS_2_ channel (*R*_MS_) is proportional to the channel distance, *R*_C_ can be extracted from the intercept of linear fitting of the total resistance as a function of channel distance if one assumes a constant contact resistance (see details in Supplementary Note 1). To ensure an identical voltage drop on the contact region, we have plotted in Fig. 2c *R*_total_ versus the channel distance (normalized by its width) at different *I*_ds_. In Fig. 2d, the extracted value of *R*_C_ rapidly drops off on increasing |*V*_ds_| whereas it dominates the total resistance at low *V*_ds_. The large variation of *R*_C_ is ascribed to the change of the Schottky profile versus *V*_ds_. This is a major issue of our devices as discussed in the following. The extracted MoS_2_ channel resistance (*R*_MS_) displays a smaller variation on increasing |*V*_ds_|. The ratio of *R*_MS_/*R*_total_ becomes saturated to be about 35% when |*V*_ds_|>0.35 V (inset of Fig. 2d). If one assumes that this ratio is still valid at |*V*_ds_|=1 V, *R*_MS_ can be estimated to be 102 kΩ for *V*_g_=+10 V and 32.8 kΩ for *V*_g_=+20 V (sheet resistance *R*_sq_∼1 × 10^5^Ω). At |*V*_ds_|=1 V and *V*_g_=+20 V, *R*_C_ approaches 2*R*_MgO_=63.7 kΩ corresponding to two intrinsic MgO barriers in series free of depletion tunnelling zone, thus giving *R*_MgO_=31.8 kΩ.

In Fig. 2e, *I*_ds_ as a function of *V*_g_ is plotted for different *V*_ds_. The transistor ON/OFF ratio can be estimated from the current ratio between *V*_g_=±20 V, which is around 2 × 10^3^. The lower ON/OFF ratio compared to the reported values^2^ is due to the influence of leakage current on *I*_ds_ (∼1.5 nA at *V*_g_=±20 V) in the OFF state because of the slight damage of contacts during wire bonding (see Supplementary Note 2). The effective field-effect mobility *μ* can be estimated by extracting the slope d*I*_ds_/d*V*_g_ from the *I*_ds_−*V*_g_ curves (Fig. 2e):


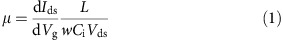


where *L* is the channel length, *w* is the channel width and *C*_i_ is the gate capacitance^2^. It is found that *μ* increases with *V*_g_ as well as *V*_ds_ (Fig. 2f). At *V*_g_=+20 V and *V*_ds_=−1 V, the mobility equals *μ*∼6 cm^2^ V^−1^ s^−1^ in close agreement with previously reported value (7 cm^2^ V^−1^ s^−1^) at 10 K for ML MoS_2_ on SiO_2_/Si substrate^28^. In this low temperature range the transport in MoS_2_ is dominated by scatterings on charged impurities^28^ or hopping through localized states^29^.

### Magnetoresistance measurements

**Figure 3:**
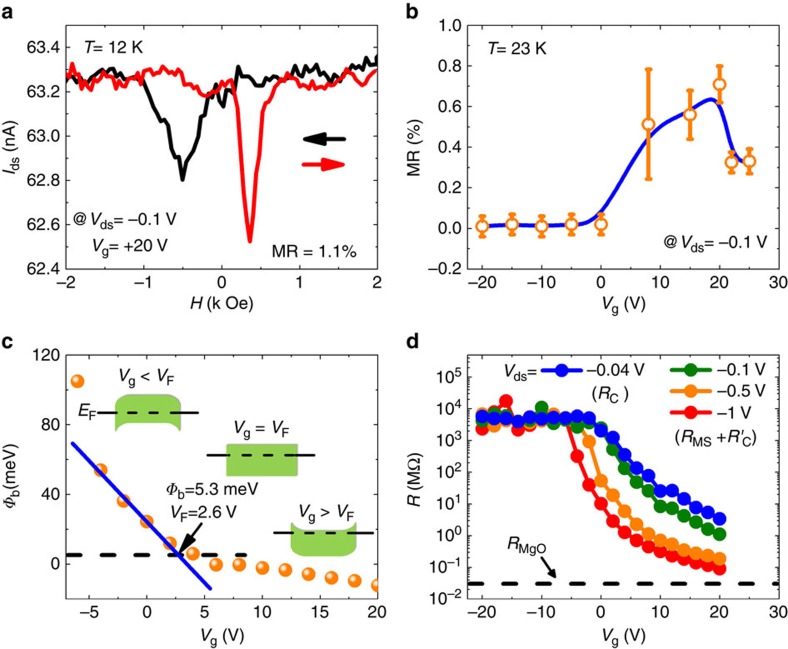
Back-gate voltage-dependent MR characterization of the device. (**a**) Magneto-resistance response of the multilayer MoS_2_-based lateral spin-valve device measured at 12 K with *V*_g_=+20 V and *V*_ds_=−0.1 V. (**b**) Back-gate voltage dependence of MR measured at 23 K with *V*_ds_=−0.1 V. The error bars have been calculated by taking account of the signal noise and the contribution of leakage current. (**c**) Back-gate voltage-dependent Schottky barrier height (Φ_b_). The deviation from the linear response at low *V*_g_ (blue solid line) defines the flat band voltage (*V*_F_) and the real Φ_b_ of Co/MgO on MoS_2_. Insets: schematics of MoS_2_ band structure with different *V*_g_. (**d**) Variation of the total resistance (*R*) as a function of *V*_g_ with different *V*_ds_ at 23 K. The error bars in **b** have been calculated by taking account of the signal noise and the contribution of leakage current.

We now focus on the key results of this paper about the MR experiments on MoS_2_. These are performed at relatively low bias (|*V*_ds_|<0.15 V) in order to avoid inherent hot-electrons spin depolarization mechanisms. Figure 3a displays the recorded magneto-current curve at 12 K with *V*_ds_=−0.1 V and *V*_g_=+20 V. A clear spin-valve signal is observed characterized by a larger current flowing in the parallel (P) state at high field and a smaller current in the quasi-antiparallel (AP) magnetic state at low field. The MR ratio can be calculated from (*I*_P_−*I*_AP_)/*I*_AP_ × 100% to be about 1.1%. This result constitutes a clear demonstration of an electron spin transport through the conduction band of MoS_2_ in a lateral geometry. We have carefully checked the angle dependence of MR, leakage current and electrode resistance to rule out any possible spurious effects on MR such as charged impurities in MoS_2_, spin transport in the Si substrate or anisotropic MR effect of Co electrodes (see Supplementary Note 3). In addition, micromagnetic simulations prove a comparable coercivity field as observed in our experimental configuration (see Supplementary Note 4). Reproducible results have been obtained on several samples and no spin signal can be found in the control non-magnetic samples (see Supplementary Note 3). All of these confirm that the MR results originate from spin transport from MoS_2_.

A very interesting feature is the particular back-gate voltage dependence of MR near the optimal condition for spin-injection/detection. Figure 3b displays MR versus *V*_g_ showing a characteristic maximum MR signal at a gate voltage of *V*_g_=+20 V. In order to clarify this point, one should first understand the effect of *V*_g_ on the transport properties. As shown in the inset of Fig. 3c, the back-gate mainly plays two roles. One is to modulate the Fermi level (*E*_F_) inside the MoS_2_ bandgap yielding a change of the carrier density in the channel^2^. The second role is to modify the SB profile and width. This scenario can be supported by the measurement of the back-gate dependence of the SB height (Φ_b_) of Co/MgO on MoS_2_, as shown in Fig. 3c (see Supplementary Note 5). It is noted that all Φ_b_ values are extracted from the measurements between 180 K to 240 K and with *V*_ds_ from −0.4 V to −1 V. Here, we can identify two regions for the variation of Φ_b_ versus *V*_g_. For *V*_g_<+2.6 V, that is when the depletion layer is thick, the thermionic emission dominates and this results in a large linear increase of Φ_b_ on the negative *V*_g_. However, for *V*_g_>+2.6 V, the tunnel current through the thin SB impinges on the linearity of Φ_b_. The real value of Φ_b_ for Co/MgO on MoS_2_ is obtained at the point of the onset of the deviation (+2.6 V) equalling thus 5.3 meV in good agreement with the value reported for Co/Al_2_O_3_ (2.5 nm) on multilayer MoS_2_ (ref. 24). This analysis would provide the SB height only when the thermal activation is dominant, that is, below +2.6 V. Above this value, the analysis can still be performed, but it does not lead to the interpretation of SB height, since tunnelling is dominant. In Fig. 3d, we have plotted the total resistance versus *V*_g_ for different *V*_ds_. As mentioned above, the resistance is mainly attributed to the contact resistance *R*_C_ at *V*_ds_=−0.04 V. At *V*_ds_=−1 V, the contribution from *R*_MS_ can reach 35% in *R*_total_ when the depletion region is much reduced. At large negative *V*_g_ when *E*_F_ is far away from the bottom of the conduction band, the resistance is rather high and does not vary with *V*_ds_ certainly preventing any spin transport in MoS_2_. For positive *V*_g_, the channel and contact resistance both decrease rapidly with *V*_g_ when *E*_F_ moves close to the conduction band. From −20 V to +20 V, the MoS_2_ channel resistance decreases much faster than the contact resistance (5 × 10^4^ times for *V*_ds_=−1 V versus 1.2 × 10^3^ times for *V*_ds_=−0.04 V).

**Figure 4:**
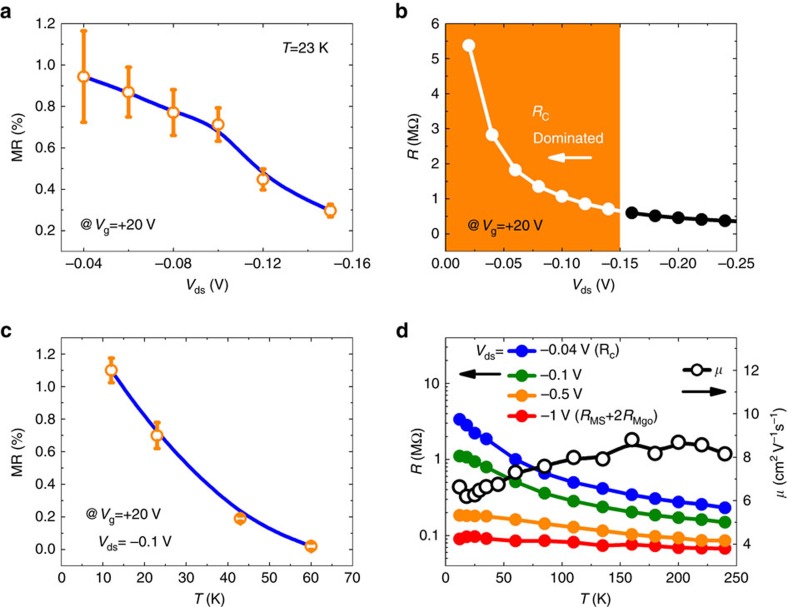
Drain-source bias and temperature-dependent MR characterization of the device. (**a**) *V*_ds_ dependence of MR measured at 23 K with *V*_g_=+20 V. (**b**) The total resistance (*R*) of the device versus *V*_ds_. The area with orange colour indicates the *V*_ds_ range where the total resistance is dominated by the contact resistance. (**c**) Temperature dependence of MR measured with *V*_g_=+20 V and *V*_ds_=−0.1 V. (**d**) Temperature dependence of the total resistance (*R*) with different *V*_ds_ and the MoS_2_ channel mobility *μ* (*V*_g_=+20 V, *V*_ds_=−1 V). The error bars in **a**,**c** have been calculated by taking account of the signal noise and the contribution of leakage current.

The bias dependence of MR measured with *V*_g_=+20 V at 12 K is displayed in Fig. 4a. It is found that the MR ratio decreases with the increase of bias |*V*_ds_|. When |*V*_ds_| is larger than 0.15 V, MR almost disappears. We note that the total resistance also decreases rapidly with the increase of |*V*_ds_| (Fig. 4b), especially in the range |*V*_ds_|<0.15 V where *R*_C_ is considered to be dominant as mentioned above. This indicates that the observation of MR could be related to the large contact resistance introduced to overcome the impedance mismatch at Co/MgO/MoS_2_ interface. In Fig. 4c, we display the temperature (*T*) dependence of MR acquired with *V*_g_=+20 V and *V*_ds_=−0.1 V: the MR rapidly decreases with *T*. When *T*>60 K, MR almost disappears. This thermal behaviour could be also associated to the variation of *R*_C_ versus *T* if one assumes a constant spin polarization at Co/MgO interface^30^. In Fig. 4d, the total resistance as a function of *T* is plotted for different *V*_ds_ conditions. It appears obviously that the resistance at *V*_ds_=−0.04 V (*R*_C_ dominant) decreases rapidly when *T*>60K, while the resistance with *V*_ds_=−1 V (*R*_MS_+2*R*_MgO_) decreases more slowly with *T*. Therefore, a strong correlation between the contact resistance and MR is also revealed from the temperature dependence of MR. In Fig. 4d, we also show the temperature variation of mobility derived at *V*_g_=+20 V and *V*_ds_=−1 V. It is noted that the mobility of MoS_2_ channel shows a slight increase with temperature up to 200 K, which could be due to the scattering from the charged impurities^28^. Supplementary data for MR measurements (temperature, bias and back-gate dependence) can be found in Supplementary Note 6.

### Spin diffusion length

**Figure 5:**
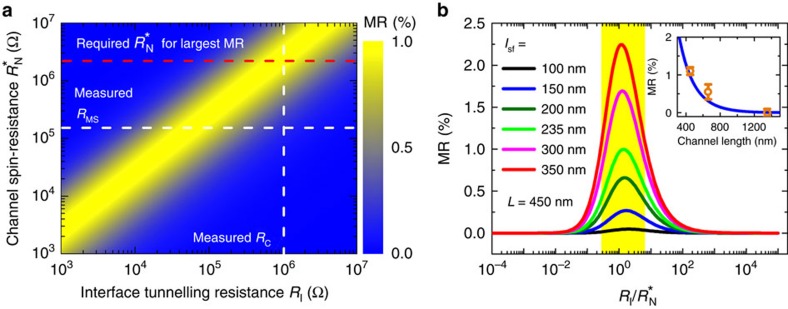
Calculation of MR with spin diffusion theory. (**a**) Calculated MR of FM/I/MoS_2_/I/FM structure as a function of the interface tunneling resistance (*R*_I_) and the channel spin-resistance (*R*_N_*). (**b**) Calculated MR as a function of the ratio *R*_I_/*R*_N_* with different spin diffusion length (*l*_sf_). Inset: theoretical calculated MR versus channel length (blue line) and experimentally observed MR between different electrodes (E1–E2, E2–E3 and E1–E3). The error bars have been calculated by taking account of the signal noise and the contribution of leakage current.

In order to explain our experimental results, we have calculated the MR from the standard theory of spin-injection adapted to lateral devices with tunnelling injectors^20^,^31^,^32^. The expected MR is calculated versus the characteristic resistances which are the tunnelling interface resistance (*R*_I_) and the channel spin-resistance (*R*_N_*=*R*_sq_˙*l*_sf_/*w*) (ref. 31) for a lateral spin-valve in the so-called ‘open' geometry (that is, the injected spin may diffuse freely in the channel outwards both contact regions)^32^. The generic formula is given by:


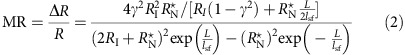


where *γ* is the tunnel spin polarization (injector and detector), chosen to be 0.5. (The spin polarization *γ* is deduced from the tunnel magnetoresistance (TMR) of a magnetic tunnel junction with amorphous Al_2_O_3_ barrier by *γ*=[TMR/(2+TMR)]^1/2^=0.5 with TMR=70%. We assume that the MgO tunnel barrier is amorphous on MoS_2_). *L* is the channel length (450 nm) and *l*_sf_ is the spin diffusion length (SDL), found to be close to 235 nm from refined analyses (detailed hereafter). The channel resistance *R*_N_ possesses a certain relationship with its spin-resistance *R*_N_* according to *R*_N_=*R*_N_*·*L*/*l*_sf_, and this makes *R*_N_ close to *R*_N_* when *L*≈*l*_sf_. As shown in Fig. 5a, one can observe a theoretical maximum value of MR of about 1% in a narrow window when the spin-dependent tunnel resistance *R*_I_ is almost equal to the channel spin-resistance *R*_N_*. This condition corresponds to a perfect balance between the spin-injection rate (1/*R*_I_) and the spin relaxation rate (1/*R*_N_*) giving a same maximum of MR for the whole range of *R*_I_ and *R*_N_*. To better demonstrate the relationship between *R*_I_ and *R*_N_*, we have plotted in Fig. 5b the MR as a function of the ratio *R*_I_/*R*_N_* for different *l*_sf_. The maximum MR increases with *l*_sf_ due to the evanescent exponential prefactor describing the spin-memory loss scaling with exp(−*L*/*l*_sf_), however always being localized around *R*_I_/*R*_N_*=1. From this optimal condition, a larger *R*_I_ would reduce the rate of spin-injection compared to the spin-flip rate and consequently reduce the spin-accumulation in MoS_2_. On the contrary, a larger *R*_N_* gives rise to a spin-backflow process by which the spin would relax in the ferromagnet (Co) and resulting in a reduced injected spin-current and MR from the optimum condition of equality between the tunnel contact resistance and channel spin-resistance (see ref. 32 and Supplementary Note 7).

Assuming the balance condition *R*_I_=*R*_N_*, the spin diffusion length (*l*_sf_) estimated from formula (2) for a 1% MR is close to 235 nm. This appears as a lower bound because of the relative high spin-polarization *γ* (0.5) chosen for Co/MgO. Other tunnelling processes than direct tunnelling would give rise to small MR by several orders of magnitude. In particular, sequential tunnelling processes via interface states (IS) between MgO and MoS_2_, like observed in Co/Al_2_O_3_/GaAs structures^33^ and responsible for spin-accumulation amplification, would be detrimental for MR. (In a spin injection/transport/detection experiments, the level of spin accumulation generated in MoS_2_ through spin-injection is generally reduced by the presence of intermediate states^33^. This would result in an overall reduction factor of (*R*_MgO_)^2^·*R*_MS_*/(*R*_SC_)^3^ even in the case of infinite spin-relaxation time on these IS. Here, *R*_MgO_ is the MgO barrier resistance (about 30 kΩ), *R*_MS_* is the spin-resistance of MoS_2_ (*R*_sq_˙*l*_sf_/*w*=20–60 kΩ) and *R*_SC_ is the Schottky resistance (MΩ).) This excludes a sequential tunnelling to form the interface resistance and emphasizes that a direct tunnelling process through one MgO and Schottky composite barrier should be taken into account. Another important conclusion is that an *l*_sf_ of 235 in multilayer MoS_2_ at low temperature is already ten times larger than the value predicted in ML MoS_2_ taking into account the DP spin-depolarization mechanism^14^. To strengthen that point, the inset of Fig. 5b displays the characteristic MR versus channel distance acquired between different electrodes (E1–2, E2–3 and E1–3) together with the simulation curve obtained for *l*_sf_=235 nm. It fingerprints the distance-dependence of the spin-injection/detection process beyond other spurious effects. The exponential decay of MR is in good agreement with the simulated MR versus the channel distance. This validates the estimated long spin diffusion length at least larger than 200 nm in our 6ML MoS_2_. From the extracted mobility of MoS_2_ channel (*μ*=6 cm^2^ V^−1^ s^−1^) at 12 K (*V*_g_=+20 V, *V*_ds_=−1 V), one can estimate the spin lifetime *τ*_sf_ to be 46 ns from *τ*_sf_=*l*_sf_^2^/(2*D*)=*l*_sf_^2^*e*/(2*μk*_B_*T*), where *D* is the diffusion constant. Remarkably, this spin lifetime is one order of magnitude larger than the electron spin relaxation time recently measured in ML MoS_2_ by optical Kerr spectroscopy^34^. Since the multilayer MoS_2_ possesses an indirect band gap restricting the ability to observe spin relaxation by optical means, the electric spin-injection/detection method provides an efficient alternate method to probe quantitatively the spin relaxation mechanisms in such compound.

## Discussion

**Figure 6:**
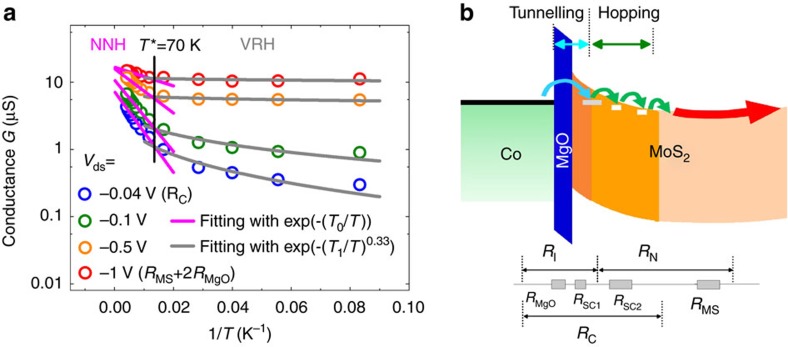
Evidence of hopping transport in the contact region. (**a**) Arrhenius plot of the temperature dependent conductance (symbols) at different *V*_ds_ from Fig. 4d and the fitting results by different hopping models (grey and pink lines). Two hopping regimes are clearly separated by T* (vertical line). (**b**) Band diagram of the Schottky contact region of the MoS_2_ device. The device can be divided into three regions. The direct tunnelling region consists of the MgO tunnelling barrier (*R*_MgO_) and one part of Schottky contact (*R*_SC1_) taken as a whole. The second region is in the tail part of depletion layer where electrons transport in a hopping behaviour (*R*_SC2_). The third region is the region where electrons either transport in the MoS_2_ channel by a hopping behaviour or transport in the MoS_2_ conduction band (*R*_MS_), depending on the carrier density.

As mentioned above, an optimized MR occurs at the balance condition of a tunnel contact resistance approaching the channel spin-resistance. It seems however impossible to perfectly fulfil such condition in our device at low *V*_ds_ bias. As shown in Fig. 5a, in the best MR situation, the MoS_2_ channel resistance (*R*_MS_) is estimated to be about 150 kΩ. The contact resistance in the investigated range of bias and temperature lies in the MΩ range, well beyond the characteristic threshold, and should then exclude any MR. How may one then reconcile with the standard spin-injection model? To clarify that point, let us focus on the *T*-dependence of the conductance as a fingerprint of the electronic hopping process involved in the transport. Figure 6a displays the Arrhenius plot of the *T*-dependence conductance at different *V*_ds_. It becomes obvious that the charge transport may be described by two distinct mechanisms in the respective high and low temperature regimes, with a characteristic threshold at T*∼70 K. For *T*>T*, the transport is dominated by the nearest-neighbour hopping (NNH) with a conductivity varying like *G*∼exp(−*T*_0_/*T*). For *T*<T*, the conductance can be fitted by a 2D variable-range hopping (VRH) equation according to *G*∼exp[−(*T*_1_/*T*)^0.33^]. Such characteristic *T*-dependence has been observed in many low-dimensional systems and is a signature of hopping transport via localized states^29^,^35^,^36^. In MoS_2_ system, it is reported that the sulphur vacancies can introduce localized donor states inside the bandgap^29^. The two temperature regions for the different hopping regimes are even more pronounced at small |*V*_ds_| (0.04 V) when the contact resistance dominates the total resistance. This highlights a transport dominated by hopping in the contact region more than in the MoS_2_ channel by itself.

In this scenario, we propose that the contact region may be constituted by three different zones (Fig. 6b): (i) the chemical MgO tunnel barrier, (ii) the strongly depleted zone underneath MgO (SC1) playing the role of an additional composite tunnel barrier and (iii) the tail of the MoS_2_ depletion zone where the electronic conduction is ensured by hopping mechanism (SC2). When one considers an inhomogeneous spin-channel made of two parts: a depletion part ‘*D*' of length *t* and a semi-infinite channel part ‘*B*', the effective channel spin-resistance reads 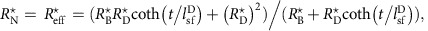 where 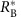 is the ‘bulk' spin-resistance of the channel and 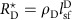 (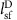 is the spin diffusion length in the depletion region). For thin depletion case (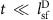), like considered here, 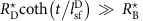, leads to 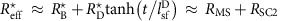.

The observation of MR at low bias goes in favour of an impedance matching at the level of the contact region, by acting with back-gate voltage, however without preventing spin injection and spin transport in the MoS_2_ channel. The impedance matching is achieved when the ratio between the tunnelling injector resistance *R*_I_ (*R*_MgO_+*R*_SC1_) and the channel effective spin-resistance (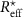) close to *R*_N_ (*R*_SC2_+*R*_MS_) approaches unity from small to large values depending on which part of MoS_2_ consists in the SC1 or SC2 regions. At low bias, the impedance matching in our Co/MgO/MoS_2_ devices is then only possible thanks to the high resistivity region (SC2) in the channel. Since the maximum MR is expected at about equal value of *R*_I_ and 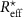, the non-linear variation of the observed MR versus *V*_g_ reflects the balance ratio between *R*_I_ and 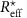. When *V*_g_ increases from +8 V to +20 V, due to the shift of *E*_F_ in the MoS_2_ band gap, the faster decay of the MoS_2_ channel resistance (*R*_MS_) compared to the tunnel injector resistance (*R*_I_) contributes to the enhancement of MR (Fig. 3d). One can also invoke the increase of the electron mobility (Fig. 2f) and spin-flip time. For larger *V*_g_, due to the shrinking of the Schottky depletion layer, a continuous decreasing of *R*_SC2_ cannot fulfil anymore the balance condition between *R*_I_ and 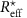, and results in the drop of MR. When increasing the bias or the temperature, the electrical field^37^ or thermal activation energy^38^ can favour electron hopping via localized states, especially for the variable-range hopping process and can effectively reduce *R*_SC2_. This explains the drop of MR with increase of *V*_ds_ and *T* is also due to the deviation of the impedance balance condition.

In conclusion, we have demonstrated the electrical spin injection and detection through the conduction band of a 450 nm long, 6ML thick MoS_2_ channel. From the systematic studies of the bias, temperature and back-gate voltage dependence of MR, it is found that the hopping via localized states in the contact depletion region plays a key role to keep the balance condition between the interface tunnelling resistance and the channel resistance, which is mandatory for the observation of the two-terminal MR. Moreover, the electron spin-relaxation is found to be greatly suppressed in the multilayer MoS_2_ channel for an in-plane spin injection geometry. The underestimated long spin diffusion length (∼235 nm) could open a new avenue for spintronic applications using multilayer TMDs.

## Methods

### Nano-device fabrication

The MoS_2_ flakes were exfoliated from a bulk crystal (SPI Supplies), using the conventional micro-mechanical cleavage technique, onto a clean SiO_2_ (100 nm)/n++-Si substrate. First e-beam lithography (Raith-150) was performed to define the four electrodes on the selected flake. Then the sample was introduced into a molecular beam epitaxy system to deposit the FM electrodes, which consists of 2 nm MgO, 10 nm Co and 10 nm Au. After deposition and lift-off, a second e-beam lithography procedure was used to define the four large pads for electrical connection. Then Ti(10 nm)/Au(190 nm) was thermally evaporated in a PLASSYS MEB400s system for the large pads. After lift-off, the device was annealed in the vacuum at 200 °C for 1 h followed by the coverage of 10 nm MgO protection layer. To check the thickness of MoS_2_ flake and the distance of channel, we have performed atomic force microscopy characterization in tapping mode on the device. In order to precisely extract the flake thickness, Gaussian fitting of the distribution of height has been employed.

### Magneto-transport measurements

The magneto-transport measurements have been performed in a cryostat varying temperature from 12 to 300 K with a maximum magnetic field of 4kOe. For the device presented in the main text, in order to reach a well-defined AP magnetic configuration, a magnetic field was applied at a 45° angle to the electrodes. Magnetic domains are then generated through the reservoir of the large triangle areas of the electrodes (Fig. 1a) before propagating towards the injector and detector regions above the MoS_2_ flake^39^ (see more micromagnetic simulations in Supplementary Note 4). For the back-gated two-terminal spin-valve measurement as described in Fig. 1d, we have used a Keithley 2400 to apply the drain-source bias *V*_ds_, and used a Keithley 6487 picoamperometer to measure the drain-source current *I*_ds_. At the same time, another Keithley 2400 was employed to apply the back-gate voltage *V*_g_.

### Data availability

The data that support the findings of this study are available from the corresponding author on request.

## 

## Additional information

**How to cite this article:** Liang, S. *et al*. Electrical spin injection and detection in molybdenum disulfide multilayer channel. *Nat. Commun.* **8,** 14947 doi: 10.1038/ncomms14947 (2017).

**Publisher's note:** Springer Nature remains neutral with regard to jurisdictional claims in published maps and institutional affiliations.

## Supplementary Material

Supplementary Information Supplementary Figures, Supplementary Notes and Supplementary References

Peer Review File
