# Supplementary material for: Electrical spin injection and detection in molybdenum disulfide multilayer channel
Source: Nat Commun. 2017 Apr 7;8:14947. doi: 10.1038/ncomms14947 (PMC5385572; doi:10.1038/ncomms14947)
Supplement: Supplementary Information — Supplementary Figures, Supplementary Notes and Supplementary References [file ncomms14947-s1.pdf]

Supplementary Note 1. Contact resistance extraction from current-voltage (I_{ds}-V_{ds}) characteristics

To correctly extract the contribution of the contact resistance (R_C) and MoS_2 channel resistance (R_{MS}), we have checked I_{ds}-V_{ds} at V_g=+10V between electrodes with different channel distances (E1-E2, E1-E3 and E1-E4), as shown in Figure 2b in the main text. At large V_{ds}, the Schottky contact resistance mainly concerns to the electrode which is reversely biased to inject the current and the contact resistance in the forward biased Schottky barrier can be neglected. Therefore, we focus on the negative V_{ds} regime corresponding to the injection of electrons from the electrode E1 to other different electrodes. Since the shape of the MoS_2 flake is triangle, different MoS_2 channel widths should also be considered.

There are two methods to extract the resistance with different channel distance. The first one is to extract the resistance at the same V_{ds}, as shown in Supplementary Figure 1a with different V_{ds}. However, the variation of *R vs.* channel distance/width is not exactly linear. This is because that there is one part of MoS_2 depletion region contributes to the contact resistance. Their resistance can be varied depending on the partial voltage dropped on it. With the increase of channel length, the voltage dropped on the MoS_2 depletion region is not the same, which introduces the non-linear behavior. The second method is to use identical I_{ds} instead of V_{ds} as shown in Supplementary Figure 1d, the linear behavior is much improved. Assuming that R_C is constant and R_{MS} is proportional to the channel distance/width, R_C can be extracted from the intercept of linear fitting of the resistance as a function of channel distance/width (Supplementary Figure 1b with identical V_{ds} method and Supplementary Figure 1e with identical I_{ds} method). In Supplementary Figure 1c and 1f, we show the extracted R_C and R_{MS} (E1-E2) as a function of V_{ds} with V_g=+10V by using identical V_{ds} and identical I_{ds} method, respectively. For the method with I_{ds}, the dependence with V_{ds} is obtained according to the I_{ds}-V_{ds} relationship between electrodes E1-E2.

With both methods, R_C decreases rapidly with increase of $|V_{ds}|$ and it dominates the total resistance at low $|V_{ds}|$. However, the method with V_{ds} overestimates R_{MS} and underestimates R_C, which results in the crossover of R_{MS} and R_C at $|V_{ds}|$=0.14V (Supplementary Figure 1c). With the method of I_{ds}, it is found that R_{MS} also decreases with increasing $|V_{ds}|$, but it is always smaller than R_C (Supplementary Figure 1f). As shown the inset of Supplementary Figure 1f, the contribution of R_{MS} in R_{total} increases with $|V_{ds}|$, and it saturates at 35% when $|V_{ds}|$ is larger than 0.35V.

In Supplementary Figure 2, we show the extraction of the contribution of R_C and R_{MS} with different channel distances at V_g=0V by using identical I_{ds} method. In Supplementary Figure 2b, the variation of *R vs.* channel distance shows a relative good linearity. In Supplementary Figure 2d, we show the extracted R_C and R_{MS} (E1-E2) as a function of V_{ds} at V_g=0V. It appears a similar behavior as V_g=+10V. R_C decreases rapidly with increase of $|V_{ds}|$ and it dominates the total resistance at low

$|V_{ds}|$. R_{MS} also decreases with increasing $|V_{ds}|$. The contribution of R_{MS} in R_{total} increases with $|V_{ds}|$ and saturates at 37% when $|V_{ds}|$ is larger than 0.25V.

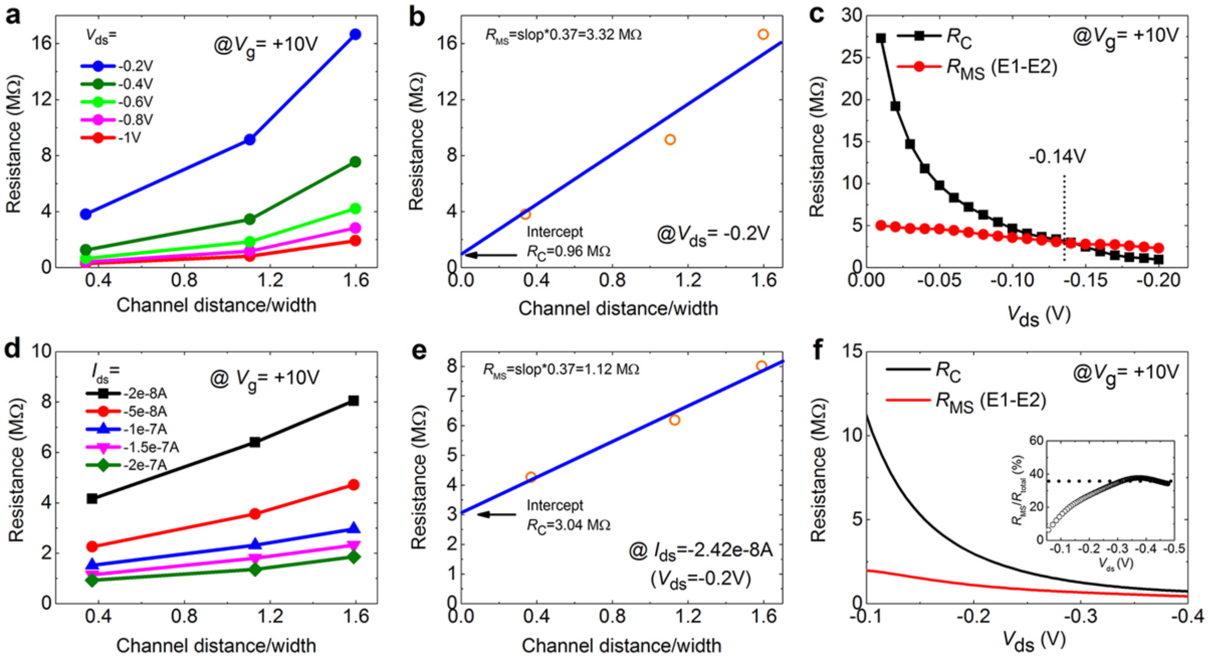

Supplementary Figure 1. (a, d) The resistance between the two electrodes versus the channel distance normalized by the width (a) with different V_{ds} and (d) with different I_{ds}. (b, e) The contact resistance can be extracted from the intercept of resistance *vs.* channel distance/width. (b) The resistance is taken with identical V_{ds}=-0.2V. (e) The resistance is taken with identical I_{ds}=-2.42e-8A, which corresponds to V_{ds} =-0.2V between E1-E2 electrodes. (c, f) The extracted contact resistance R_C and the MoS_2 channel resistance R_{MS} *vs.* V_{ds}. at V_g=+10V with (c) identical V_{ds} method and (f) identical I_{ds} method. Inset of (f): percentage of R_{MS}/R_{total} as a function of V_{ds}.

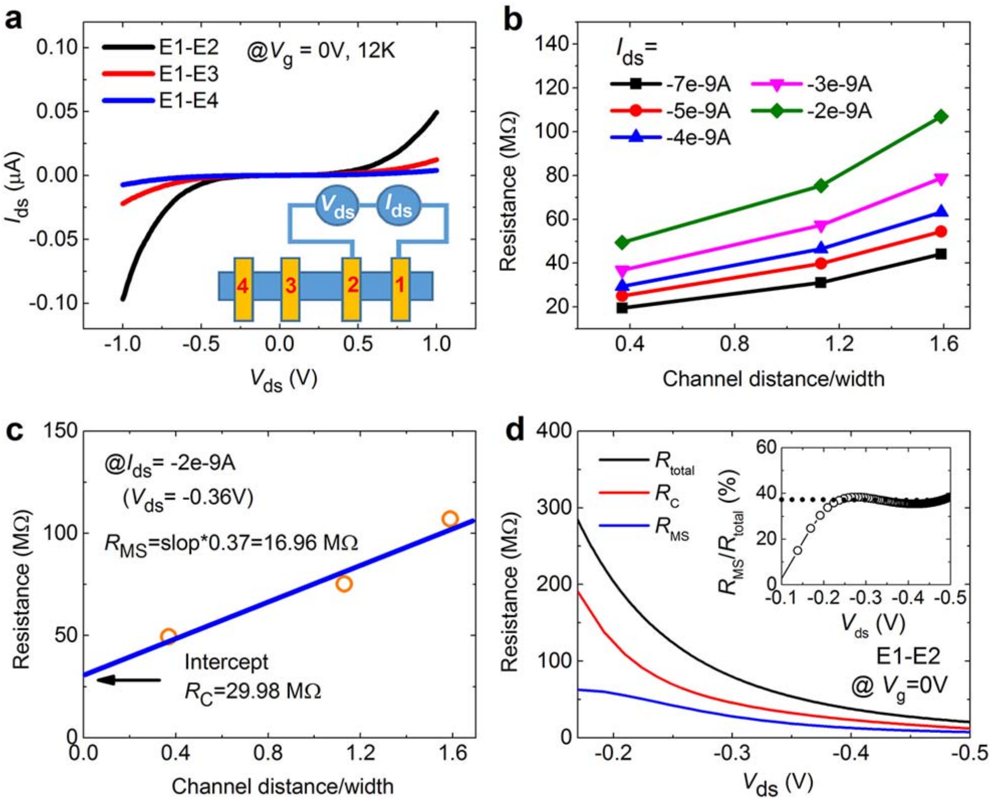

Supplementary Figure 2. (a) I_{ds}-V_{ds} characteristics measured between different electrodes at 12K with applying a back-gate voltage V_g=0V. (b) The resistance between the two electrodes versus the channel distance normalized by the width with different I_{ds}. (c) The contact resistance can be extracted from the intercept of resistance *vs.* channel distance/width. The resistance is taken with identical I_{ds}=-2e-9A, which corresponds to V_{ds} =-0.36V between E1-E2 electrodes. (d) The total resistance R_{total}, the extracted contact resistance R_C and the MoS_2 channel resistance R_{MS} *vs.* V_{ds}. at V_g=0V. Inset: percentage of R_{MS}/R_{total} as a function of V_{ds}.

Supplementary Note 2. Leakage current

During the wire-bonding procedure, unintentional damage was created on the connection pads on the Si/SiO_2 substrate, which results in a small leakage current between the electrode and Si substrate when applying a large back-gate voltage. This leakage current I_g can influence the measured drain-source current I_{ds} through the MoS_2 channel when the contact resistance becomes comparable to the leakage resistance. Supplementary Figure 3a shows the schematics of the electrical connections of the device and Supplementary Figure 3c illustrates the equivalent electric circuit considering four electrodes on the MoS_2 flake. If we assume that the leakage resistance is equal for the four electrodes R_g=R_{g1}=R_{g2}=R_{g3}=R_{g4} and MoS_2 resistance is equal between each two close electrodes R_{mos}=R_{MS1}=R_{MS2}=R_{MS3}, we can obtain:

$$I_{\mathrm{ds}} = \frac{V_{\mathrm{ds}}}{R_{\mathrm{mos}}} + \frac{V_{\mathrm{g}}}{R_{\mathrm{g}}} \left(\frac{R_{\mathrm{g}}^2 + 4R_{\mathrm{g}}R_{\mathrm{mos}} + 3R_{\mathrm{mos}}^2}{R_{\mathrm{g}}^2 + 3R_{\mathrm{g}}R_{\mathrm{mos}} + R_{\mathrm{mos}}^2} \right) - I_{\mathrm{g}} \quad (1)$$

Since R_{mos} changes a lot with V_{g}, when $R_{\mathrm{mos}} << R_{\mathrm{g}}$, $I_{\mathrm{ds}}=V_{\mathrm{ds}}/R_{\mathrm{mos}}+V_{\mathrm{g}}/R_{\mathrm{g}}-I_{\mathrm{g}}$ and when $R_{\mathrm{mos}} >> R_{\mathrm{g}}$, $I_{\mathrm{ds}}=V_{\mathrm{ds}}/R_{\mathrm{mos}}+3V_{\mathrm{g}}/R_{\mathrm{g}}-I_{\mathrm{g}}$. In Supplementary Figure 3b, we show the measured leakage current I_{g} as a function of V_{g}. It is found that the leakage current is linearly proportional to the back-gate voltage with no change for different V_{ds}, and it reaches ±5.6nA with V_{g}=±20V, respectively. Since our MR measurements is in the range of 1-100nA, the leakage current could modify the MR for the small I_{ds} case, but it does not influence the large I_{ds} case. To precisely obtain MR values, we have taken account of the leakage current when calculating MR, and put the corresponding error bars in the figures.

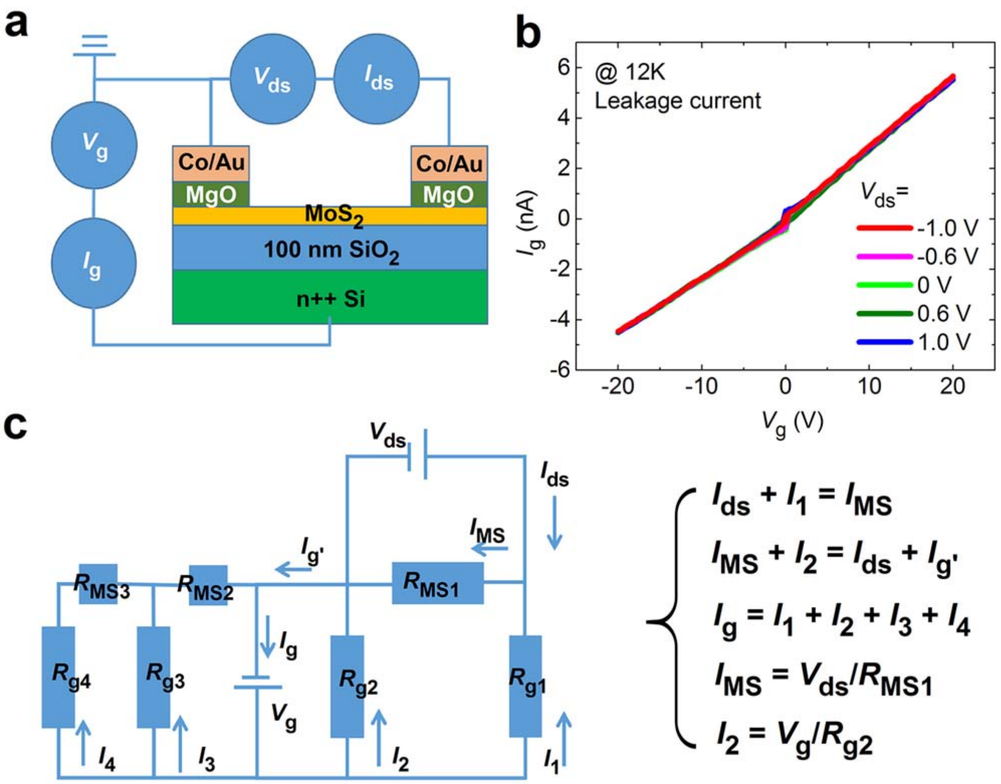

Supplementary Figure 3. (a) Schematics of the multilayer MoS_2 based lateral spin-valve device. (b) Leakage current as a function of back-gate voltage. (c) Equivalent circuit with finite leakage resistance.

Supplementary Note 3. Possible artificial effects for the spin transport

Since the measured MR ratio in our sample is small (~1%), any artificial effects related to the substrate and electrodes could affect our conclusions, and we should carefully verify their influence on the measurements.

A. *Control sample with non-magnetic contact*

To eliminate artificial effects on the magneto-transport measurement related to the MoS_2 channel, Si substrate *etc.*, we have fabricated one control sample with total non-magnetic electrodes, as shown in Supplementary Figure 4a. The thickness of the flake is identical to the device in the main text (4.3nm, 6MLs). The MoS_2 channel distance between electrodes E2 and E3 is about 400nm. Supplementary Figure 4b shows the characterization of I_{ds} as a function of V_{ds} with different V_g. The I_{ds}-V_{ds} properties of the flake is similar to the device in the main text, however we cannot get any magneto-response with V_g=+10V (Supplementary Figure 4c) or V_g=+20V (Supplementary Figure 4d). This gives a strong argument that the MR effect we measured is related to the magnetic states of electrodes.

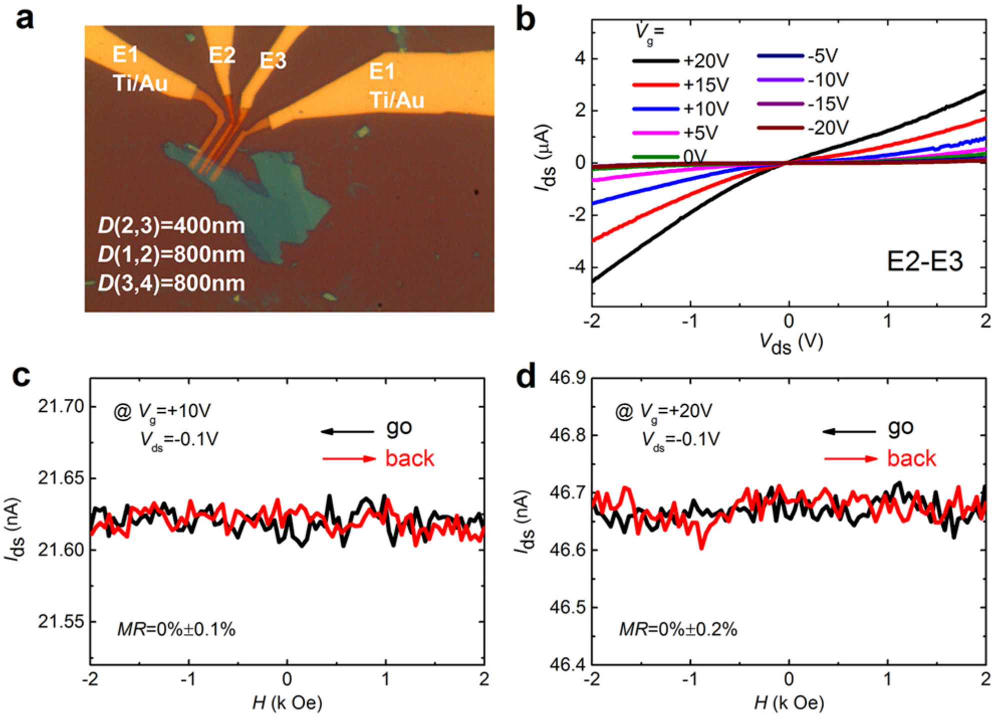

Supplementary Figure 4. (a) Optical microscopy image of the control non-magnetic device. The region of dark yellow consists of MgO(2nm)/Au(10nm) and the region of light yellow consists of Ti(10nm)/Au(190nm). (b) I_{ds}-V_{ds} characteristics between E2 and E3 measured at 12K with applying different V_g. (c) Magneto-resistance response of the device with V_{ds}=-0.1V and V_g=+10V at 12K. (d) Magneto-resistance response of the device with V_{ds}=-0.1V and V_g=+20V at 12K.

B. *Leakage current effect*

We have mentioned above that for V_g=±20V we measure about ±5.6nA leakage current. Since Si is a very good candidate for spin transport and it has been reported that spin-polarized electrons can transport for even 300μm distance in Si[1]. Therefore, it is very important to exclude the

possibility of spin transport through the bottom Si substrate instead of MoS_2 channel. If the observed MR is due to the Si substrate, we should also observe MR in negative V_g. In Supplementary Figure 5, we show the I_{ds} *vs.* H curve with negative V_g=-16V and V_{ds}=-0.1V at 20K. It is clear that we cannot observe any spin signal, which proves that the observed MR is not due to the leakage current through Si substrate.

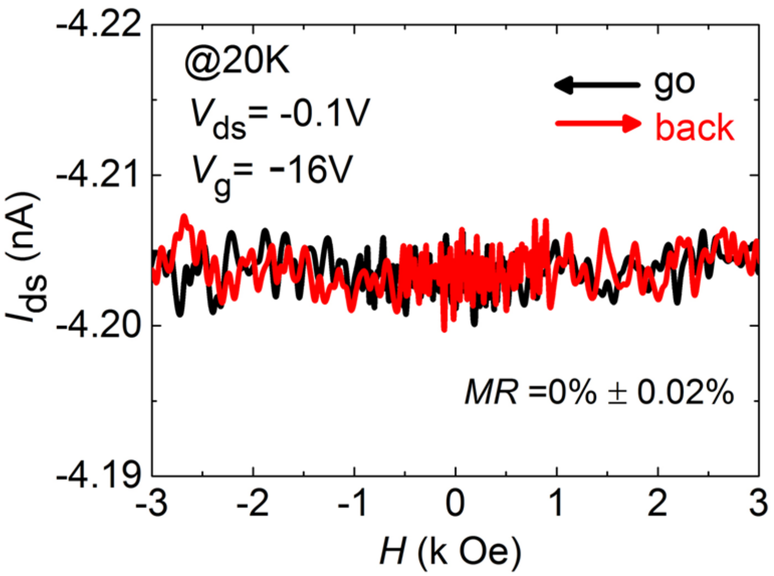

Supplementary Figure 5. Magneto-resistance response measurement with V_g=-16V to show the absence of MR signal.

C. Anisotropic magneto-resistance of electrodes

We also need to verify if the anisotropic magneto-resistance (AMR) of Co electrode could play a role for the spin-dependent transport. The resistance of Co electrode (L10μm×W300nm×H10nm) can be estimated to be about 390Ω (ρ_{Co}:117nΩ·m at 20K). If there is 1% AMR in Co electrode[2], the variation of resistance is only 3.9Ω. It is completely negligible compared to the measured variation of resistance (~16kΩ with 1% of MR). Therefore, the possibility of AMR can be excluded.

Supplementary Note 4. Micromagnetic simulation on magnetization switching of Co nano-wire

We have performed the micromagnetic simulations of the magnetization switching in Co nano-wires by using the external magnetic field. This simulation is to understand if we can obtain different coercivity with the same width of electrode but different shapes. For this purpose, we have used the Mumax3 software[3]. Two different nano-wire geometries (Supplementary Figure 6a) were

used to simulate the experimental condition (Supplementary Figure 6b, E1 and E2 electrodes). An external magnetic field was applied at 0° and 45° in respect to nano-wire easy axis in order to check the switching fields for each shape of Co nano-wire. Parameters used in the simulation were taken from the literature[4]: Exchange stiffness (20×10^{-12}Jm^{-1}), saturation magnetization (939×10^{3}Am^{-1}), first order uniaxial anisotropy constant (450×10^{3}Jm^{-3}) and the Landau-Lifshitz damping constant (0.5). The uniaxial anisotropy was set to be along the -x direction with a small component on the +y axis (10%) in order to facilitate the switching by an external magnetic field swept along the +x direction. The width of wires along the y axis was set to be 300nm corresponding to the one from the experiment. The nanowire length was set to be 7 times longer than its width and the thickness was set to 10nm. The initial magnetization state was chosen to be uniform, pointing out in the -x direction. All simulations were performed for T=0K. As a result, a distinct difference of switching field around 100Oe can be obtained for the two kinds of shape when the angle between the electrodes and field is either 0° (Supplementary Figure 6c) or 45° (Supplementary Figure 6d). The order of value is in good agreement with the experimental observed antiparallel plateau (200-500Oe) if we consider the real shape and defect pinning effect on the domain wall prorogation.

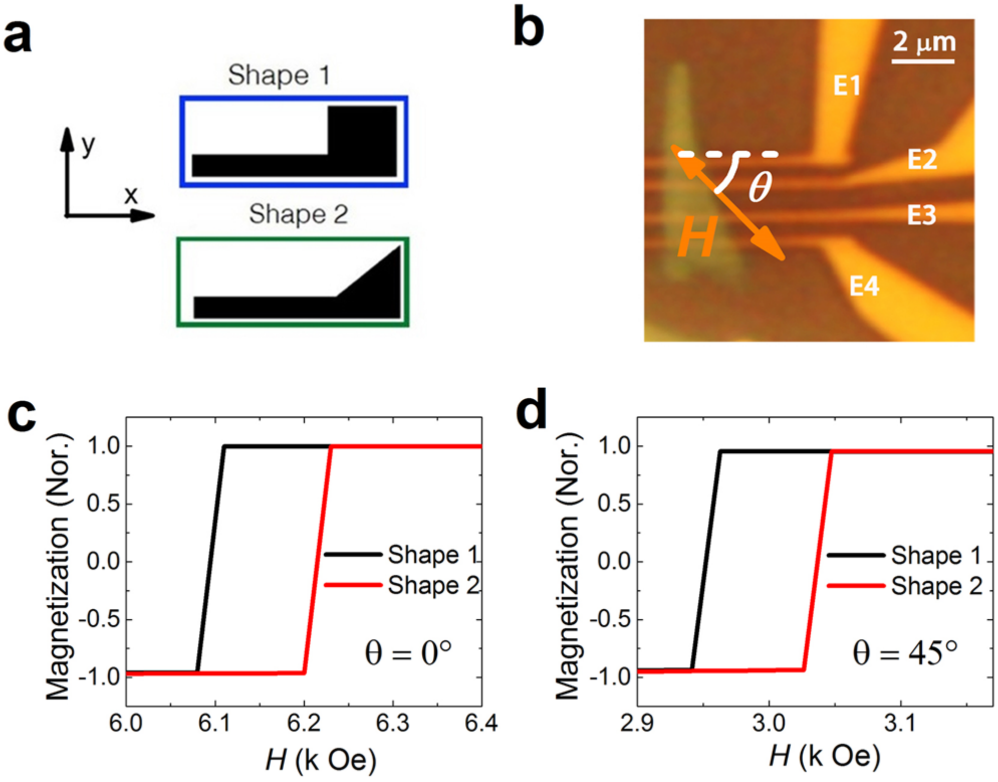

Supplementary Figure 6. (a) Two different shapes used for micromagnetic simulation. The width of wire is set to be 300nm. (b) Optical microscopy image of the device in the main text. (c) Simulated magnetization switching for the two kinds of shape when magnetic field is along 0° to the electrodes. (d) Simulated magnetization switching for the two kinds of shape when magnetic field is along 45° to the electrodes.

Supplementary Note 5. Schottky barrier height of Co/MgO on MoS_2

In order to estimate the Schottky barrier height of Co/MgO on MoS_2 (Supplementary Figure 7a), we have measured I_{ds}-V_{ds} characteristics with different V_g from 180K to 240K; in this temperature range where the thermionic emission transport mechanism through the Schottky barrier is mainly considered (Supplementary Figure 7b). We employed a two-dimensional thermionic emission equation describing the electrical transport through the Schottky barrier into the MoS_2 channel[5]:

$$I_{\mathrm{ds}} = AA^{*}T^{1.5}\exp\left[-\frac{e}{k_{\mathrm{B}}T}\left(\varPhi_{\mathrm{b}} - \frac{V_{\mathrm{ds}}}{n}\right)\right] \qquad (2)$$

where A is the contact area, A^* is the Richardson constant, e is the electron charge, k_B is the Boltzmann constant, $\varPhi_b$ is the Schottky barrier height, and n is the ideality factor. Supplementary Figure 7c shows the Arrhenius plot ($\ln(I_{ds}/T^{3/2})$ *vs.* $1000/T$) for different V_{ds}. The slopes $S(V_{ds})$ extracted from the Arrhenius plot follow a linear dependence with V_{ds}: $S(V_{ds})=-(e/1000k_B)\cdot(\varPhi_b-V_{ds}/n)$, as displayed in Supplementary Figure 7d. Then the Schottky barrier height can be evaluated from the extrapolated value at zero V_{ds} ($S_0=-(e\varPhi_b/1000k_B)$). In Supplementary Figure 7d, we can obtain a Schottky barrier height $\varPhi_b$ of 11.9meV for Co/MgO on MoS_2 with V_g=+2V. Similar procedure has been used to determine $\varPhi_b$ with different V_g, as described in the main text (Figure 3c).

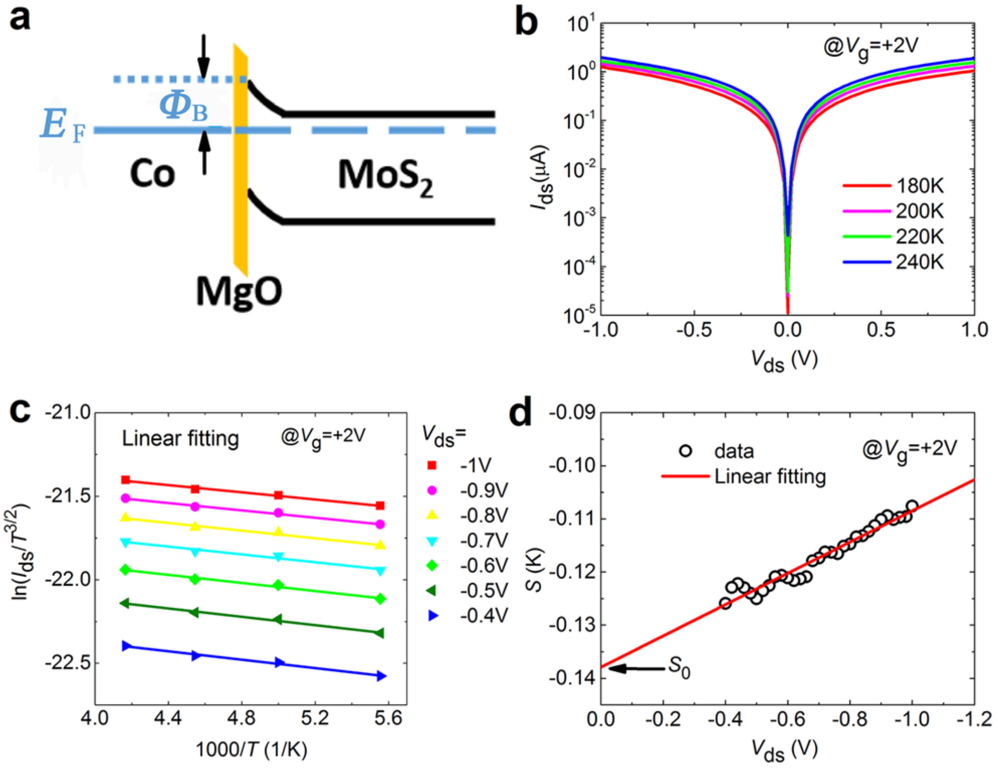

Supplementary Figure 7. (a) Schematics of the Schottky barrier height for Co/MgO contact on MoS_2. (b) I_{ds}-V_{ds} characteristics for temperatures between 180K and 240K. (c) $\ln(I_{ds}/T^{3/2})$ versus $1000/T$ at different drain-source bias

(V_{ds}), in an Arrhenius plot with linear fits in the temperature range from 180K to 240K. (d) Bias dependence of the slope (S). The slope at zero V_{ds} (S_0) is used to calculate the Schottky barrier height Φ_b.

Supplementary Note 6. Supplementary data for magnetoresistance measurements

Here we show all raw data for temperature (Supplementary Figure 8), drain-source bias V_{ds} (Supplementary Figure 9) and back-gate voltage V_g (Supplementary Figure 10) dependent MR measurements. All MR values have been corrected after considering the leakage current as mentioned in Supplementary Note 2.

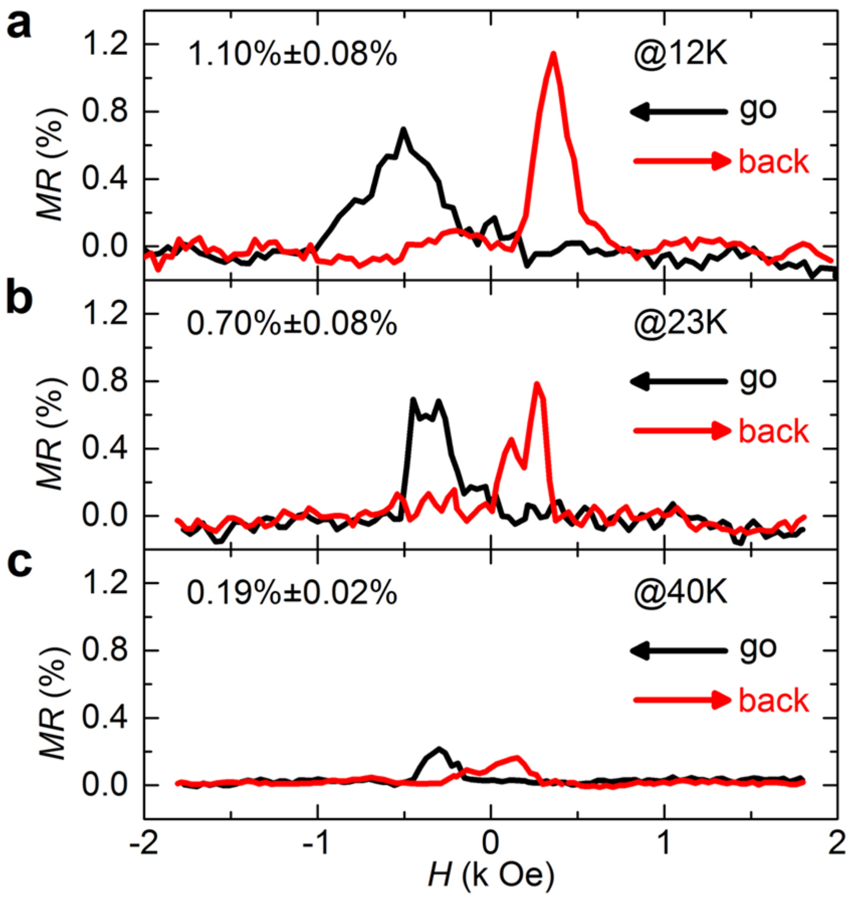

Supplementary Figure 8. Magneto-resistance response of the multilayer MoS_2 based lateral spin valve device, measured with V_g=+20V and V_{ds}=-0.1V at (a) 12K, (b) 23K and (c) 43K, respectively. The error bars have been calculated by taking account of the signal noise and the contribution of leakage current.

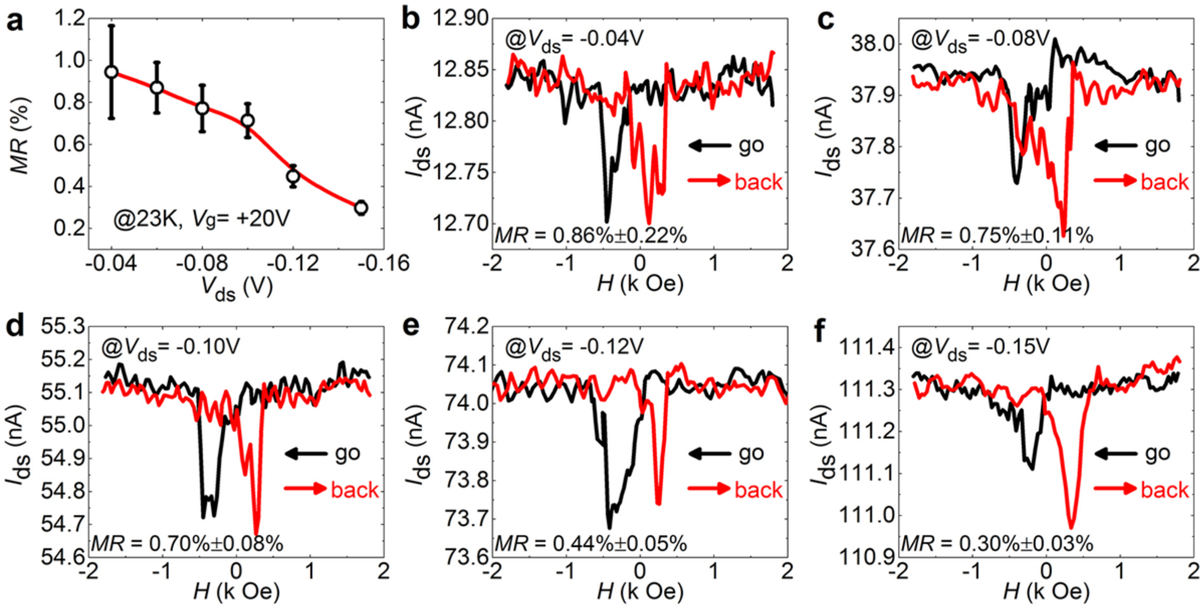

Supplementary Figure 9. V_{ds} dependence of MR. (a) MR versus V_{ds}. (b-f) Magneto-resistance response measured with V_g=+20V at 23K with V_{ds} from -0.04V to -0.15V. The error bars have been calculated by taking account of the signal noise and the contribution of leakage current.

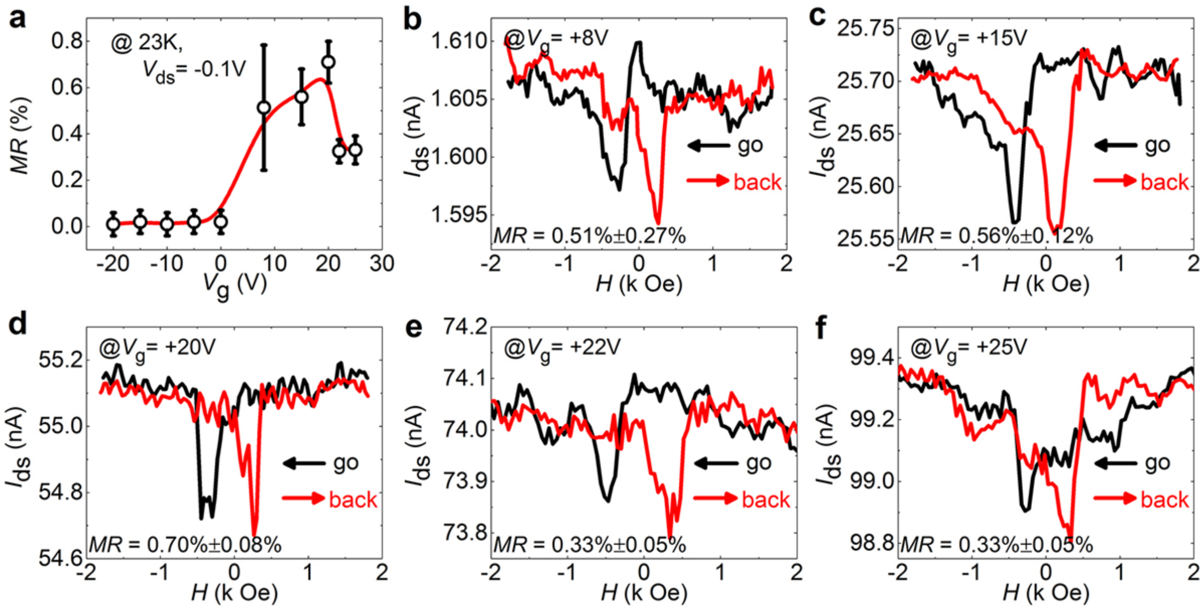

Supplementary Figure 10. V_g dependence of MR. (a) MR versus V_g. (b-f) Magneto-resistance response measured with V_{ds}=-0.1V at 23K with V_g from +8V to +25V. The error bars have been calculated by taking account of the signal noise and the contribution of leakage current.

Supplementary Note 7. Supplementary information for spin injection/detection theory

Magnetoresistance experiments between ferromagnetic source and drain like performed on MoS_2 devices are typical prototype experiments in which a spin-current is generated by electrical means in MoS_2 from a spin-dependent tunnel injector, transported in MoS_2 over several hundreds of nanometers, before being absorbed at the level of a same tunnel spin-polarized detector. We can discriminate between the 'local' geometry whereby the charge current flows in both contacts and through the channel to the so-called 'non-local' geometry whereby the spin-current diffusing out of the injector is detected at the level of the detector without the need of a charge current displacement, only by probing a change in the voltage detection. It has been shown that the two configurations give exact identical magnetoresistive signals up to a factor of 2 whatever the contact sizes and channel geometry. This was derived in a general frame of spin-diffusion relaxation model[6].

From very general arguments, the existence of a large MR requires two main conditions: 1) The injection of a large spin-current in the parallel (PA=↑↑) magnetic state from a tunnel injector necessary to overcome the impedance mismatch issue. This is generally realized by using a MgO tunnel barrier with a high spin-injection efficiency thanks to its spin-filtering property. 2) The second condition is the occurrence of a large spin-accumulation signal in the antiparallel (AP=↑↓) magnetic state necessary to obtain a large contrast of resistance between the two magnetic configurations. The second condition can be generally more difficult to fulfill because of the spin-relaxation which may occur in the channel by spin-flip processes between the spin-injection from the tunnel injector and the spin-detection processes at the level of the drain. For relative high resistive MgO tunnel barrier like used here, this second condition is more drastic than the necessary condition of a short channel distance compared to the characteristic spin diffusion length (l_{sf} or SDL) and corresponding to the standard exponential decrease exp($-L/l_{sf}$) like observed in this work. This second condition can lead to a drop of MR even in the case of a short channel distance because of electronic spin multi-reflection processes on MgO barriers.

Owing to the ensemble of these arguments, the general shape of the MR in a source-drain geometry have been largely discussed in the literatures[6-8] and its very general expression writes according to a certain sum-rule:

$$\Delta V = eJ\Delta R = 2\left(P^{\uparrow\uparrow}\Delta\mu^{\uparrow\downarrow} - P^{\uparrow\downarrow}\Delta\mu^{\uparrow\uparrow}\right) \qquad (3)$$

where P is the spin-current polarization and $\Delta\mu$ is the spin accumulation measured at a given point in the channel. Indeed, one can easily demonstrate, by simple derivation, that the previous expression is coordinate-invariant along the spin propagation in the channel from the Valet-Fert theory of diffusive spin-transport[9]. If one chooses the middle point between in the channel as the reference point, one sees that the MR is maximal for either $P^{\uparrow\downarrow} = 0$ or $\Delta\mu^{\uparrow\uparrow} = 0$ corresponding to a

symmetric device between spin injector/detector. In that sense, a symmetric system means the same contact sizes with same barrier thickness and same materials. Any asymmetry, *e.g.* like obtained in forward/reversed Schottky barrier profile may lead to a decrease of the maximal MR signal expected from its nominal largest value. Nevertheless, in the present case, the best MR experiments have been performed at very low bias from -0.04V to -0.1V where the resistance asymmetry between forward and reversed MoS_2 Schottky barriers remain small and then irrelevant for the particular MR asymmetry analyses.

On the other hand, from these whole arguments discussed here, the largest value to expect for MR and its optimization deals in several points and parameters in terms of quality of tunneling spin-injectors/detectors, and channel geometry. The tunneling spin-injectors/detectors have to be spin-selective enough and in this mind the choice of the MgO barriers is very adequate. On the other hand, the tunnel barriers must be high resistive enough to avoid the impedance mismatch issue and spin-backflow processes[7,10,11]. This is in the spirit to increase $P^{\uparrow\uparrow}$ at its maximum value of γ (tunnel spin-polarization) in the meaning that any spin-backflow process will reduce to a smaller (vanishing) value for a low resistive barrier resistance (compared to the channel spin-resistance by itself). In our situation of MgO/MoS_2 devices, the impedance matching required is ensured in a certain optimized window by a gate voltage acting on both tunnel barrier and the MoS_2 channel resistance composed of two different parts: a MoS_2 depleted region with location of hopping underneath the tunnel contacts and the 'bulk' MoS_2 part in the middle. This particular circumstance of a dual channel properties requires to revisit somehow the optimal conditions of spin injection as explained below.

The second optimization of the device in favor of a large MR can be performed on a certain increase of $\Delta\mu^{\uparrow\downarrow}$ via an optimized channel geometry design[8]. $\Delta\mu^{\uparrow\downarrow}$, which has to be maintained large in the channel and in particular at the channel middle point, represents in the AP state an equilibrium value between the rate of spin-injection and the rate of spin-orbit assisted spin-flips. By quenching any spin-flip process depending on the total volume of spin-relaxation, MR should increase in parallel to $\Delta\mu^{\uparrow\downarrow}$. In that situation, a channel characterized by a long spin-diffusion length λ_{sf} is very favorable in several points. First, a long λ_{sf} allows to relieve the exponential decrease of the MR signal due to spin-flip processes (spin-memory), and second, a long λ_{sf} is very favorable like in multilayer MoS_2 like investigated here, the rate of spin-flips being scaled-down by $1/(\rho\lambda_{\mathrm{sf}})$ in bulk material or in channel that spreads at infinity on both sides of the device. We call this as the so-called 'open-geometry' ('O'). However, when the channel is limited in its lateral sides, the rate of spin-flip decreased as $L/(\rho\lambda_{\mathrm{sf}}^2)$, where L is the distance between source and drain. We call this as the so-called 'confined-geometry' ('C'). Decreasing the distance L between the source and the drain

to a value well smaller than λ_{sf}, or conversely increasing λ_{sf}, by material engineering is a way to largely increase the MR signal.

On the other hand, for a short distance L compared to λ_{sf}, the way to increase the MR is to adapt the tunnel barrier, and in particular its thickness t_{MgO} to an optimized value of resistance close to $\rho \frac{\lambda_{\mathrm{sf}}^2}{t_{\mathrm{MoS2}}}$ where t_{MoS2} is the channel thickness. This value corresponds to an optimal situation for impedance matching and limited spin-relaxation in the MoS_2 channel.

Upon all these arguments, one can give a general expression of the local (and non-local resistance to a factor of 1/2) given below depending on the different device geometry.

$$\frac{\Delta R_{\mathrm{O}}}{R_{\mathrm{N}}^*} = \frac{8\gamma^2 R_{\mathrm{I}}^2}{\left(2R_{\mathrm{I}}+R_{\mathrm{N}}^*\right)^2 - \left(R_{\mathrm{N}}^*\right)^2 \exp\left(-\frac{2L}{\lambda_{\mathrm{sf}}}\right)} \exp\left(-\frac{L}{\lambda_{\mathrm{sf}}}\right) \quad (4)$$

for an open geometry with symmetric contacts and

$$\frac{\Delta R_{\mathrm{C}}}{R_{\mathrm{N}}^*} = \frac{8\gamma^2 R_{\mathrm{I}}^2}{\left(R_{\mathrm{I}}+R_{\mathrm{N}}^*\right)^2 - \left(R_{\mathrm{I}}-R_{\mathrm{N}}^*\right)^2 \exp\left(-\frac{2L}{\lambda_{\mathrm{sf}}}\right)} \exp\left(-\frac{L}{\lambda_{\mathrm{sf}}}\right) \quad (5)$$

for a closed geometry with symmetric contacts, where R_{I} is the tunnel barrier resistance (in Ohm) and R_{N}^* is the channel spin-resistance (in Ohm).

The expressions for asymmetric tunnel barriers are the same except to replace R_{I} by $\frac{R_{\mathrm{I}}^1+R_{\mathrm{I}}^2}{2}$ and $(R_{\mathrm{I}})^2$ by $(R_{\mathrm{I}}^1\, R_{\mathrm{I}}^2)$ which have the effect to decrease the signal from its nominal value obtained for $R_{\mathrm{I}}^1 = R_{\mathrm{I}}^2$ (R_{I}^1 and R_{I}^2 are the two tunnel resistances). However, the observed decrease is generally small. All these expressions correspond to the most general aforementioned expression (3) for MR.

In the case where the channel region admits an inhomogeneous character like *e.g.* composed of two mixed regions (a MoS_2 depleted region of length *t* and a 'bulk' channel region spreading up to infinity), the spin-resistance of the channel R_{eff}^* has to be recalculated according to $R_{\mathrm{eff}}^* = \frac{R_{\mathrm{B}}^* R_{\mathrm{D}}^* \coth\left(\frac{t}{l_{\mathrm{sf}}^{\mathrm{D}}}\right) + (R_{\mathrm{D}}^*)^2}{R_{\mathrm{B}}^* + R_{\mathrm{D}}^* \coth\left(\frac{t}{l_{\mathrm{sf}}^{\mathrm{D}}}\right)}$, where R_{B}^* is the 'bulk' channel spin-resistance, R_{D}^* is the spin-resistance of the depletion zone and l_{sf}^D is its characteristic spin diffusion length. A large R_{D}^* compared to R_{B}^*, obtained at a certain extend depletion zone (*t*) leads to a significant increase of R_{eff}^* and then to the impedance matching with the MgO barrier resulting sizeable MR value (threshold for impedance mismatch).

Supplementary References:

1. Huang, B. Q., Monsma, D. J. & Appelbaum I. Coherent spin transport through a 350-micron-thick Silicon wafer. *Phys. Rev. Lett.* **99**, 177209 (2007).
2. Gil, W., Görlitz, D., Horisberger, M. & Kötzler, J. Magnetoresistance anisotropy of polycrystalline cobalt films: Geometrical-size and domain effects. *Phys. Rev. B* **72**, 134401 (2005).
3. Vansteenkiste, A., Leliaert, J., Dvornik, M., Helsen, M., Garcia-Sanchez, F. & Van Waeyenberge, B. The design and verification of mumax3. *AIP Advances* **4**, 107133 (2014).
4. Beaujour, J.-M. L., Chen, W., Kent, A. D. & Sun, J. Z. Ferromagnetic resonance study of polycrystalline cobalt ultrathin films. *J. Appl. Phys.* **99**, 08N503 (2006).
5. Anwar, A., Nabet, B., Culp, J. & Castro, F. Effects of electron confinement on thermionic emission current in a modulation doped heterostructure. *J. Appl. Phys.* **85**, 2663-2666 (1999).
6. Jaffrès, H., George, J.-M. & Fert, A. Spin transport in multiterminal devices: Large spin signals in devices with confined geometry. *Phys. Rev. B* **82**, 140408(R) (2010).
7. Fert, A. & Jaffrès, H. Conditions for efficient spin injection from a ferromagnetic metal into a semiconductor, *Phys. Rev. B* **64**, 184420 (2001).
8. Laczkowski, P. *et al.* Enhancement of the spin signal in permalloy/gold multiterminal nanodevices by lateral confinement. *Phys. Rev. B* **85**, 220404(R) (2012).
9. Valet, T. & Fert, A. Theory of the perpendicular magnetoresistance in magnetic multilayers. *Phys. Rev. B* **48**, 7099-7113 (1993).
10. Schmidt, G., Ferrand, D., Molenkamp, L., Filip, A. & van Wees, B. J. Fundamental obstacle for electrical spin injection from a ferromagnetic metal into a diffusive semiconductor. *Phys. Rev. B* **62**, R4790-R4793 (2000).
11. Rashba, E. I. Theory of electrical spin injection: Tunnel contacts as a solution of the conductivity mismatch problem. *Phys. Rev. B* **62**, R16267-R16270 (2000).
